# The Effect of Myosin Light Chain Kinase on the Occurrence and Development of Intracranial Aneurysm

**DOI:** 10.3389/fncel.2018.00416

**Published:** 2018-11-13

**Authors:** Yaying Song, Peixi Liu, Zongwei Li, Yuan Shi, Jun Huang, Sichen Li, Yingjun Liu, Zhijun Zhang, Yongting Wang, Wei Zhu, Guo-Yuan Yang

**Affiliations:** ^1^Department of Neurology, Ruijin Hospital, Shanghai Jiao Tong University School of Medicine, Shanghai, China; ^2^Department of Neurosurgery, Huashan Hospital of Fudan University, Shanghai, China; ^3^Neuroscience and Neuroengineering Research Center, School of Biomedical Engineering, Med-X Research Institute, Shanghai Jiao Tong University, Shanghai, China; ^4^Shanghai Key Laboratory of Hypertension, Department of Hypertension, Shanghai Institute of Hypertension, Ruijin Hospital, Shanghai Jiao Tong University School of Medicine, Shanghai, China

**Keywords:** aneurysm, myosin light chain kinase, phenotype switch, proteomic, smooth muscle cell

## Abstract

Myosin light chain kinase is a key enzyme in smooth muscle cell contraction. However, whether myosin light chain kinase plays a role in the occurrence or development of intracranial aneurysms is not clear. The present study explored the function of myosin light chain kinase in human intracranial aneurysm tissues. Five aneurysm samples and five control samples were collected, and smooth muscle cells (SMCs) were dissociated and cultured. A label-free proteomic analysis was performed to screen the differentially expressed proteins between aneurysm and control samples. The expression and function of myosin light chain kinase in aneurysms were examined. We found that 180 proteins were differentially expressed between the aneurysm and control samples, among which 88 were increased and 92 (including myosin light chain kinase) were decreased in aneurysms compared to control tissues. In a model of the inflammatory environment, contractility was weakened and apoptosis was increased in aneurysm SMCs compared to human brain SMCs (*p* < 0.05). The knock down of myosin light chain kinase in human brain SMCs caused effects similar to those observed in aneurysm SMCs. These results indicated that myosin light chain kinase plays an important role in maintaining smooth muscle contractility, cell survival and inflammation tolerance.

## Introduction

Intracranial aneurysm (IA), as a cerebrovascular disease, has an annual incidence of approximately 1–2% and is characterized by the ballooning of the intracerebral artery with high mortality due to vascular rupture (Rinkel, 2008; Brown and Broderick, 2014). Ongoing studies characterize the process of IA formation as hemodynamic stress, thrombus formation, extracellular matrix degradation, inflammatory responses and structural changes, including SMC phenotypic modulation and consequently apoptosis (Frosen et al., 2012; Frosen, 2014). The current treatment for IA mainly involves clipping for surgical intervention and endovascular coiling based on its special characteristics. Experimental aneurysm studies have mainly focused on endothelial cells, such as the autologous endothelial cell-seed stent (Zhu et al., 2008) and enhanced aneurysm neck endothelialization by erythropoietin-induced endothelial progenitor cell stimulation (Liu et al., 2016). In addition, endothelial injury may result from the reaction and migration of SMCs to the tunica intima (Etminan and Rinkel, 2016). Evidence has also indicated that SMCs might be involved in neo-intima formation in a ligation model (Yuan et al., 2017). Moreover, the dysfunction of endothelial cells and the apoptosis or phenotypic modulation of SMCs could accelerate the progression of aneurysms.

The phenotypic modulation or dedifferentiation of SMCs could be caused by genetic defects or stimulated by stress, which are involved in many vascular diseases (Frosen, 2014; Liu et al., 2015). SMCs alter the synthetic phenotype in the medial layer, thereby weakening the vessel wall, disturbing hemostasis and finally collapsing the vascular structure. Previous studies using microarray (Yu et al., 2014), mRNA sequencing analysis (Kleinloog et al., 2016), and proteomics (Wang et al., 2016) have implicated a group of reported genes, mRNAs and related proteins involved in the proliferation, migration, and apoptosis of SMCs. Previous studies have demonstrated that the injury or death of SMCs by inflammation results in the occurrence and development of aneurysms. Furthermore, SMCs are the main components of the walls of brain vessels and aneurysms (Kondo et al., 1998; Owens, 2007). Because IASMCs were isolated from aneurysm walls, these cells have been considered as a cell model to understand the phenotype and function of SMCs in the formation, progress and rupture of aneurysms (Dai et al., 2006; Bygglin et al., 2011).

The aim of the present study was to explore the role of SMC phenotypic modulation in IA pathogenesis. We used proteomic analysis to explore the specific protein functions in IA pathology. IASMCs were isolated to further explain the results of clinical samples and investigate the role of aneurysm SMCs in IA progression.

## Materials and Methods

### Ethics Statement

This study was approved by the Institutional Review Board (IRB) and the Ethics Committee of Huashan Hospital, Fudan University, China. Each participant provided written informed consent to participate in this study.

### Tissue Collection and Label-Free Proteomic

Ten patients with IA who underwent microsurgical clipping were enrolled, and the samples were collected. The control group included patients who underwent STA surgery, followed by unavoidable clipping. Among the 10 samples, 5 were used for label-free proteomics, 2 were used for immunostaining, and 3 were used for western blotting. These 10 samples were frozen immediately at -80°C for further experiments. In addition to these 10 samples, another 4 samples were used for IASMC isolation, of which 3 samples were successful. These 10 samples were all frozen immediately at -80°C for further experiments. Label-free proteomics was commissioned by the Shanghai Branch of the Chinese Academy of Sciences.

### IASMC Isolation and Culture

Intracranial aneurysm tissues were obtained from the brain tissues of 3 IA patients during microsurgery. The modified isolation protocol was based on a previously described study (Bygglin et al., 2011; Huang et al., 2017). After clipping, the samples were immediately collected into DMEM supplemented with 5% penicillin/streptomycin. The tissue segments were washed three times with phosphate-buffered saline (PBS) supplemented with 1% penicillin/streptomycin. The surrounding connective tissue was separated, and the endothelial cell layer was scratched gently. Then, the tissues were dissected into 1 mm × 1 mm fragments that were evenly arranged on a dish and incubated in a humidified atmosphere of 5% CO_2_ and 95% air at 37°C in a medium comprising DMEM supplemented with 20% fetal bovine serum and 1% penicillin/streptomycin. After 1–2 weeks, the cells grown from the IA explants reached semiconfluence and were subcultured in smooth muscle cell medium (Lonza, Basel, Switzerland) after trypsinization. Control human brain vascular SMCs were obtained from ScienCell Research Laboratories (Carlsbad, CA, United States) and cultured in smooth muscle cell medium. We used the markers anti-myosin-11 and SMA to identify the SMCs.

### Immunostaining of Human Tissue Samples

The samples were fixed in 4% paraformaldehyde and embedded in paraffin. Following antigen retrieval by microwaving in pH 6.0 citrate buffer, the sections were incubated with anti-myosin-11 (1:50 dilution; Santa Cruz Biotechnology, Santa Cruz, CA, United States), anti-MLCK (1:200 dilution; Abcam, Cambridge, MA, United States), and anti-SMA (1:100 dilution; Abcam), followed by an immunofluorescent secondary antibody. The brain sections were then stained with DAPI and mounted. Images were taken at different magnifications with a microscope (Leica, Solms, Germany).

### MLCK siRNA Interference

Human brain vascular smooth muscle cells (HBVSMCs, Zhongqiao Xinzhou Company, Shanghai, China) were seeded onto six-well plates. Then, 100 nmol/L siRNA was diluted with 250 μl of Opti-MEM (Gibco, Carlsbad, CA, United States) and incubated for 5 min. Next, 10 μl of Lipofectamine^®^ 2000 (Invitrogen, Carlsbad, CA, United States) was diluted with 250 μl of Opti-MEM and incubated for 5 min. The above samples were mixed, generating a final 500 μl Opti-MEM solution, and incubated for 20 min. This medium was added to 2 ml of SMCM (ScienCell, Carlsbad, CA, United States) and incubated with the cells for 6 h. After changing to normal medium, the cells were cultured for 72 h. The interference was detected by real-time PCR and Western blot analysis. The following siRNA sequences were used: si-*mlck*: 5′-3′ CCTGCTTTCATTTTGCCCCC, TCACAAGGCTGAAAGTCCCC.

### Cell Viability Assay

Smooth muscle cells were plated in triplicate in 96-well plates at 4 × 10^3^ cells per well. After 24 h, the cells were treated with TNF-α at different doses, including 0, 0.5, 5, 10, 20, and 40 ng/ml for 2 h. The cck-8 assay kit was prepared according to the manufacturer’s instructions and then 100 μl was added to each well and incubated for 2 h at 37°C. The absorbance was measured at 450 nm with a spectrometer.

### RNA Extraction and Real-Time PCR

Total RNA was isolated from the cells using TRIzol reagent (Invitrogen) according to the manufacturer’s instructions. The integrity of the RNA was quantified by using the NanoDrop 1000 spectrophotometer (Thermo Fisher Scientific, UT, United States). The reverse transcription reaction and real-time PCR were performed according to the manufacturer’s instructions for the ABScript II cDNA Synthesis Kit (ABclonal, Wuhan, China) and the SYBR Premix ExTaq II Kit (Takara, Dalian, China) by a real-time PCR system (7900HT, ABI). No non-specific amplification was observed based on the dissociation curve. Glyceraldehyde 3-phosphate dehydrogenase (GAPDH) was used as an internal control. The data were analyzed using the comparison Ct (2^-ΔΔC_t_^) method and expressed as a fold change relative to the respective control. The following sequences were used for qPCR primers: ACTA2: 5′-3′ TTGAGAAGAGTTACGAGTTG, AGGACATTGTTAGCATAGAG; MYL9: 5′-3′ CGGGCCACATCCAATGTCTT, CCATGTTTGAGGATGCGGGT; MLCK: 5′-3′ GGGGACTTTCAGCCTTGTGA, CTGCTTCGCAAAACTTCCTTCT; and CNN1 5′-3′ GGCCCAGAAGTATGACCACC, CCGTCCATGAAGTTGTTGCC.

### Western Blotting Analysis

Equal amounts of protein per lane (30 μg) were subjected to electrophoresis on a 12% SDS-PAGE gel. The proteins were electrotransferred onto a polyvinylidene difluoride membrane (PVDF, Millipore, Billerica, MA, United States). The membrane was blocked with 5% non-fat dry milk/0.1% Tween-20 in Tris-buffered saline for 1 h at room temperature. Thereafter, the membrane was incubated with different primary antibodies, including rabbit anti-MLCK (1:5000 dilution, Abcam), rabbit anti-SMA (1:200 dilution, Abcam) and rabbit anti-cleaved caspase-3 (1:1000 dilution, Cell Signaling Technology). Subsequently, the membrane was treated with secondary antibody for 2 h at room temperature. Immunoblots were probed using enhanced ECL substrate (Thermo, Rockford, IL, United States). The chemiluminescence level was recorded using an imaging system (Bio-Rad, Hercules, CA, United States). The results were normalized to β-actin.

### Calcium Fluo-4 AM Assay and Contraction Study

Smooth muscle cells were preloaded with the calcium-sensitive fluorophore Fluo-4 AM in extracellular solution for 40 min at room temperature as described in a previous study (Granata et al., 2017). Then, the cells were washed for 30 min at room temperature. Intracellular calcium flux was monitored as a time series, and the acquisition rates were 1 frame every 0.1 ms over 1 min using a Leica confocal microscope before and after the addition of pilocarpine. To detect intracellular calcium release, the SMCs were incubated with calcein. A concentration of 20 mM calcein was used for stimulation. Calcium flux was detected in HBVSMCs (Zhongqiao Xinzhou Company, Shanghai, China), si-*mlck*SMCs, and IASMCs before and after TNF-α stimulation. Five cells were randomly selected from a field of view, and the fluorescent trace was analyzed using ImageJ pro plus software (Media Cybernetics, Bethesda, MD, United States).

### Statistical Analysis

All data were expressed as the means ± SE. ANOVA with Student–Newman–Keuls multiple comparisons posttest was used for gene expression. Comparisons between two groups were made by Student’s *t*-test. A *p* < 0.05 was considered significantly different. Statistical analysis was carried out with Prism GraphPad 6. Each *in vitro* experimental group was repeated three times, and the experiments were performed separately three times. These outcomes are from data averaged statistically.

## Results

### Sample Collection and Label-Free Proteomic Analysis

**FIGURE 1:**
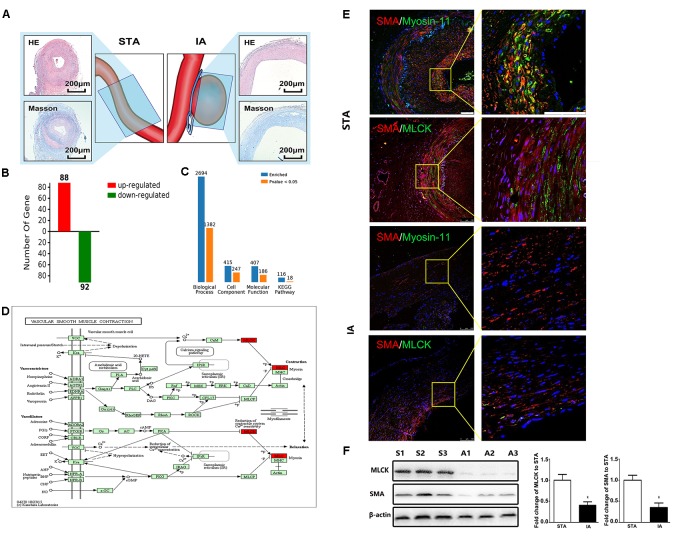
Basic information from label-free proteomics and verification in tissue samples. **(A)** IA and STA illustration with HE and Masson staining show the specimen location for label-free proteomics. **(B)** Significant differences in protein levels between IA and STA. **(C)** GO ontology analysis between IA and STA. **(D)** KEGG pathway mapping of smooth muscle cells (SMCs). **(E)** Double staining for SMA/myosin-11 and SMA/MLCK in STA and IA, with 2× magnification for a closer view. **(F)** Western blot and quantification studies of MLCK and SMA in the tissue sample. S1–S3 represents proteins from three different STA samples, and A1–A3 represents IA samples. STA, superficial temporal artery; IA, intracranial aneurysm.

Intracranial aneurysm (*n* = 5) and STA (*n* = 5) groups without significant differences in sex, age, or risk factors were compared. More information is shown in Supplementary Table 1. The location of sample collection was shown by HE and Masson staining (Figure 1A). The aneurysm wall showed less muscle and collagen compared with the STA wall. Among all 1908 proteins identified from 15426 peptides, 180 significantly differentially expressed proteins between IA and STA were identified, among which 88 were upregulated and 92 were downregulated in IA (Figure 1B). GO ontology analysis classified the proteins into “Molecular function,” “Cellular component,” and “Biological process” subcategories. Further protein expression data were obtained from the KEGG Mapper Pathway to illustrate changes in biological processes (Figure 1C). MLCK encodes a regulatory light chain of myosin II, known as myosin light-chain kinase, which was downregulated in IAs. In the vascular smooth muscle contraction pathway, MLCK was involved in the mechanism of smooth muscle contraction (Figure 1D).

### MLCK Is Downregulated in Both Tissue Samples and Primary IASMCs

**FIGURE 2:**
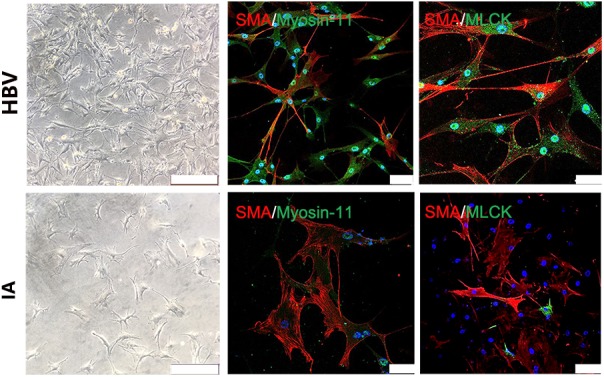
Myosin light chain kinase primary SMCs. Bright field images show IASMCs with more irregular morphology than HBVSMCs. Double staining for SMA/myosin-11 and SMA/MLCK showed that IASMCs were less or weakly positive compared with HBVSMCs. Data are presented as the means ± SD, ^∗^*p* < 0.05, bar = 100 μm. HBVSMC, human brain vascular smooth muscle cell; IASMC, intracranial aneurysm smooth muscle cell.

Tissue samples from STA and IA were double stained for SMA and MLCK. MLCK showed higher expression in the IA group, and SMA showed weaker expression in the STA group compared with the IA group. Myosin-11 is a marker of mature SMCs, and co-staining with SMA showed a reduction in contractile SMCs. Magnification also showed low SMA expression, which indicated the loss of SMCs in IA tissues. The SMC density showed a significant decrease in the IA sample, indicating that degeneration occurred in the aneurysm wall (Figure 1E). The IA samples showed a more significant downregulation of MLCK than STA, consistent with findings in proteomics. SMA was also downregulated, which suggested that functionally mature SMCs were lost in the aneurysm wall (Figure 1F). To examine whether the downregulation of MLCK in IA tissue has a similar effect in SMCs, we further isolated primary SMCs from IA walls (IASMCs). IASMCs were morphologically modulated and appeared as spider-like cells with the loss of mature SMC markers. These morphologic changes demonstrated that ductility decreased when stress suddenly increased. Cell viability was also weakened throughout the culture process. IASMCs appeared irregularly shaped and showed weak positivity for SMA, MLCK and myosin-11 compared with HBVSMCs. The results of immunostaining indicated that the IASMCs were in an immature state (Figure 2).

### MLCK Downregulation in Primary IASMCs and Its Impact on SMC Function

**FIGURE 3:**
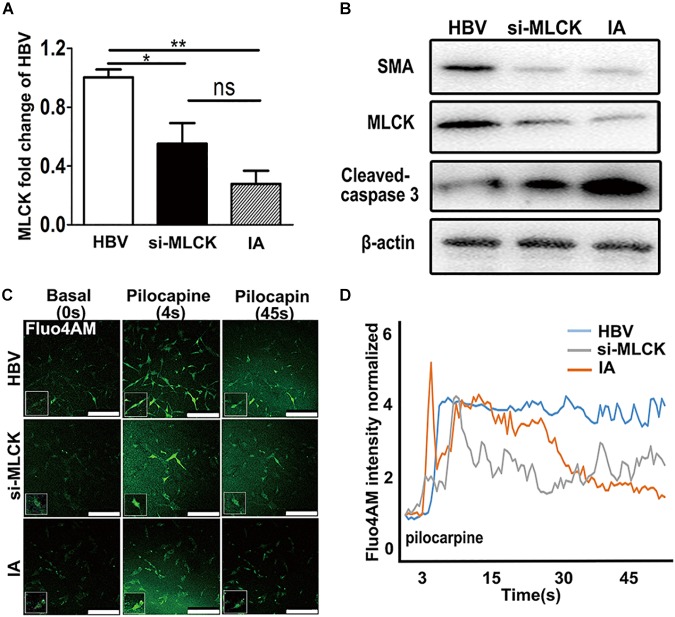
Both si-*mlck*HBVSMCs and IASMCs show weak proliferation and contraction. **(A,B)** MLCK expression in HBVSMCs, si-*mlck*HBVSMCs and primary IASMCs was detected at the mRNA and protein levels. **(C)** Ca^2+^ flux measured by Fluo-4 AM loading and the intensity in HBVSMCs, si-*mlck*HBVSMCs, and IASMCs at a basal level (0 s), upon stimulation with pilocarpine (4 s) and at 45 s after stimulation. **(D)** Quantification of contractility measured as the fluorescence change in the cell surface area of HBVSMCs, si-*mlck*HBVSMCs, and IASMCs. The relative mRNA level was normalized to GAPDH. Data are presented as the means ± SD, ^∗^*p* < 0.05, ^∗∗^*p* < 0.01. HBVSMC, human brain vascular smooth muscle cell; IASMC, intracranial aneurysm smooth muscle cell; si-*mlck*SMC, HBVSMCs with interfered *mlck.*

To investigate the function of SMCs, we used siRNA to interfere with *mlck* in HBVSMCs to mimic the genetic defects in IASMCs. The level of *mlck* showed 50% interference, with no significant difference compared with IASMCs (Figure 3A). Western blot results showed that the expression of the mature SMC markers MLCK and SMA was decreased in both IASMCs and si-*mlck*SMCs compared with HBVSMCs. We then used cleaved caspase-3 to detect apoptosis. The results indicated that with the loss of mature SMC markers, SMCs probably progressed to apoptosis (Figure 3B).

We detected cell contractility in these three groups to further investigate the role of MLCK during SMC contraction. Pilocarpine is a cholinergic agent that can activate the cholinergic receptor. SMCs can respond to the stimulus and contract. When the SMCs were incubated with calcine, intracellular calcium release was detected. The influx of calcium is not the same as “contraction,” but the cyclic calcium wave in response to an agonist may reflect cell contractility. SMCs with lower MLCK expression showed reduced contractility in response to stimulation with the cholinergic agent pilocarpine. The local cell morphology of these three groups was also different when simulated by pilocarpine. HBVSMCs showed a spindle shape, and cells with low expression of MLCK showed a more stellate pattern (Figure 3C). The intracellular calcium responses of HBVSMCs were different from those of both si-*mlck*SMCs and IASMCs. HBVSMCs generated cyclic calcium waves, whereas si-*mlck*SMCs and especially IASMCs did not generate propagating calcium waves and returned abruptly to basal levels (Figure 3D).

### Genetic Loss of MLCK Impacts the Inflammatory Responses of SMCs

**FIGURE 4:**
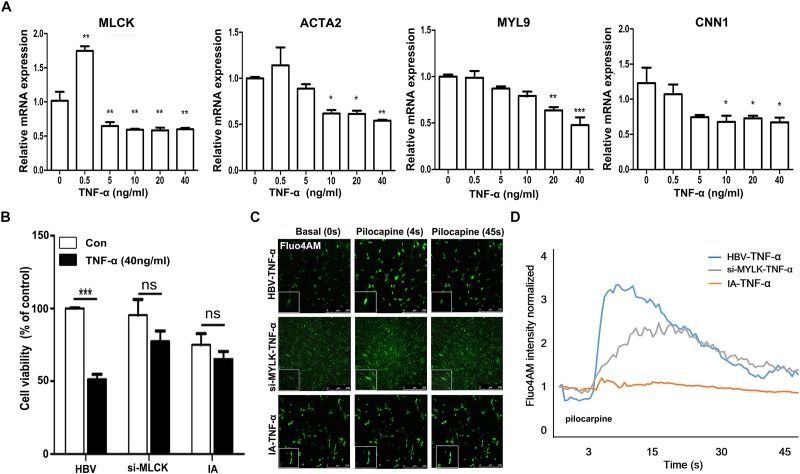
Genetically downregulated MLCK affects cell function more with the stimulation of TNF-α. **(A)** HBVSMCs were treated with a gradient dose of TNF-α and the levels of the mature SMC markers MLCK, ACTA2, MYL9, and CNN1 were detected. **(B)** All three kinds of cells were detected for cell viability by cck-8. **(C)** Ca^2+^ flux measured by loading Fluo-4 AM. **(D)** Quantification of contractility measured as the fluorescence change in the cell surface area of HBVSMCs, si-*mlck*SMCs, and IASMCs. The relative mRNA level was normalized to the control. Data are presented as the means ± SD, ^∗^*p* < 0.05, ^∗∗^*p* < 0.01, ^∗∗∗^*p* < 0.001. HBVSMC, human brain vascular smooth muscle cell; IASMC, intracranial aneurysm smooth muscle cell; si-*mlck*SMC, HBVSMCs with interfered *mlck*.

To mimic the IA environment, we administered TNF-α to explore the proliferation and contractility of SMCs. The mRNA levels of four mature SMC genes, *mlck*, *acta2*, *myl9*, and *cnn1*, were detected after treatment with different doses of TNF-α (Figure 4A). We found that mature SMC marker expression was reduced by stimulation with higher doses of TNF-α. The expression of mature SMC markers, such as ACTA2, MYL9, CNN1, and MLCK, was largely decreased after treatment with 40 ng/ml of TNF-α compared with that after treatment with other TNF-α concentrations in cultured SMCs. Therefore, we used a concentration of 40 ng/ml to treat HBVSMCs, si-*mlck*SMC, and IASMCs. Interestingly, HBVSMCs stimulated with TNF-α showed lower cell viability than IASMCs and si-*mlck*SMCs. Thus, the effect of *mlck* deficiency at the genetic level on cell viability may differ from that on inflammation (Figure 4B). We redetected cell contractility to further investigate the impact on cell viability. All of the cells showed reduced contractility in response to pilocarpine stimulation, with altered cell morphology. However, HBVSMCs appeared as slim fusiform but still responded to pilocarpine. For IASMCs, the cell morphology was not clear and showed little response to stimulation. The si-*mlck*SMCs appeared as short rod shapes but with a weaker response to calcium stimulation (Figure 4C). It was clearly observed that with the reduction of MLCK, the contractility was reduced in all three groups, and IASMCs could hardly generate calcium waves, and cells that genetically lacked MLCK seemed more vulnerable to inflammatory attacks (Figure 4D).

## Discussion

**FIGURE 5:**
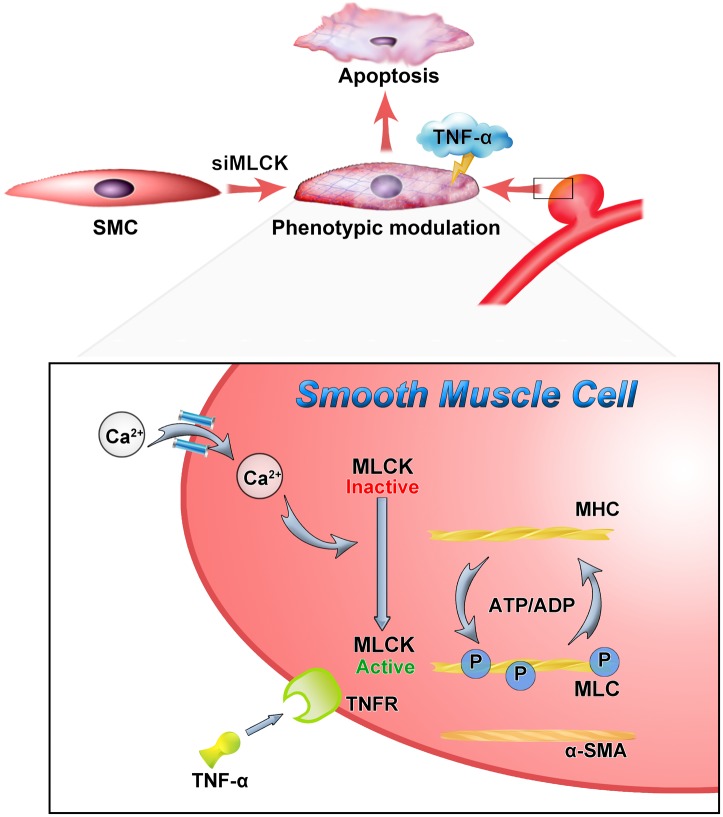
Down-regulation of MLCK weaken the contractile function and with vulnerably reaction to inflammation. The loss of MLCK expressed as phenotypic modulation and calcium dependent phosphorylation was further disturbed. In the TNF environment, the morphologically altered smooth muscle is weaker and more susceptible to apoptosis.

This study observed the effects of MLCK downregulation in IA samples using a label-free proteomic analysis. SMCs with downregulated MLCK underwent phenotypic modulation and were vulnerable to apoptosis when stimulated with TNF-α. MLCK expression was also decreased in SMCs isolated from IA walls (Figure 5). Normal SMCs with downregulated MLCK showed disturbed contractile function and were vulnerable to inflammation. In the label-free proteomic analysis, proteins that play a role in IA pathology were found, which mirrored the results found in previous studies using iTRAQ (Wang et al., 2016). Using a DNA microarray, Chen et al. (2014) found that *myh11, acta2, mlck, and my19* were differentially expressed genes associated with VSMC contraction (Chen et al., 2014). Our study focused on SMCs and highlighted the impact of losing MLCK on IA formation.

After MLCK was downregulated, the contraction of SMCs was disturbed. SMCs undergo phenotypic modulation with less contractile gene expression and show a pro-inflammatory, dedifferentiated phenotype (Owens, 1998; Owens et al., 2004; Yoshida and Owens, 2005). Phenotypic modulation and eventual degeneration have been considered to promote the formation and progression of IA. IASMCs were first isolated by Bygglin et al. (2011), and the present study was the first to detect the functions of IASMCs and the contractile gene *mlck*. IASMCs were morphologically modulated and appeared as spider-like cells with the loss of mature SMC features. A study of rabbit aneurysm models showed large and stellate cells with long cytoplasmic extensions (Dai et al., 2006). The IASMCs no longer displayed tightly arranged spindle-like cells, and the morphology was replaced by a sparse disordered form whereby the cells dissociated from each other (Merei and Gallyas, 1980). This morphological change suggests a decrease in ductility when placed under sudden stress.

Smooth muscle cell contraction and relaxation are affected not only by how much Ca^2+^ is infused but also by oxidative stress and inflammation directly or indirectly (Carvalho-de-Souza et al., 2013). Previous studies have demonstrated that the myosin light chain phosphatase regulatory light chains were associated with myosin, whose contractions were dictated by the stimulation of myosin ATPase activity (Murthy, 2006). Calcium increased and bound to calmodulin, and the calcium/calmodulin complexes then combined with MLCK, leading to MLC phosphorylation. We also showed the morphological changes and detected the contractility of IASMCs and HBVSMCs with downregulated *mlck* in Figures 3, 4. With increasing Ca^2+^ levels, calcium could bind to calmodulin and lead to MLC phosphorylation. In an aneurysm environment, the intracellular concentration of Ca^2+^, as a second messenger, decreased. Therefore, the phosphorylation induced by calcium was also downregulated. Ca^2+^/calmodulin-dependent MLCK phosphorylated MLC is essential for the initiation of smooth muscle contractions. Sustained MLC phosphorylation could be induced by Ca-independent MLCK. Thus, with downregulated MLCK, MLC phosphatase activity may also be downregulated. MLCK and MLC phosphatase activity can coregulate the relaxation of cells (Murthy, 2006). SMC relaxation can result from the activation of TGR5 by the inhibition of the RhoA/Rho kinase pathway. MLC phosphorylation via Ca^2+^-independent MLCK can sustain G protein activation and regulate the inhibition of MLC phosphatase (Rajagopal et al., 2013).

The *mlck* gene is prominently differentially expressed in IA compared with control arteries. In our experiment, MLCK expression could also be decreased by inflammatory stimulation, and SMCs showed lower viability with a higher dose of TNF-α. During intracranial inflammation, MLCK expression was decreased in SMCs. Additionally, MLC phosphatase activity might also be downregulated (Murthy, 2006). It is largely unknown whether MLCK causes or results from the phenotypic and functional modulation of SMCs. Our results suggested that MLCK might promote SMC dysfunction; however, this is the first study to support this hypothesis. Although TNF-α could suppress the expression of mature SMC genes, normal SMCs could retain the ability of contraction under certain inflammatory conditions (Ali et al., 2013). When inflammation occurred in SMCs lacking MLCK, the contractions were profoundly suppressed, thus explaining why some IA failed to maintain stability throughout the lifetime of an individual.

Intracranial aneurysm pathology also involves apoptosis, which leads to the weakening intima and media of aneurysm walls. Inflammatory factors, such as TNF-α, could trigger an inductive signal in the initiation of apoptosis (Jamous et al., 2007; Sprague and Khalil, 2009; Ait-Oufella et al., 2011). TNF-α and IL-1 are the most important pro-inflammatory factors, which are often used to induce a focal inflammatory response. Numerous studies have demonstrated that TNF-α increases and plays a critical role in the occurrence and development of aneurysms. Therefore, we used TNF-α to mimic the environment of an aneurysm (Ali et al., 2013; Aoki et al., 2014; Starke et al., 2014). Under an inflammatory environment, smooth muscle relaxation was inhibited, which was caused by soluble guanylyl activity (Rajagopal et al., 2015). SMC dysfunction or apoptosis were considered the destructive events in a ruptured IA. Our results indicated that the loss of MLCK in SMCs could lead to apoptotic progression and promote injury in a pro-inflammatory environment. The continuous loss of SMCs and functional synthesis of collagen and matrix components resulted in aneurysm enlargement and rupture (Frosen, 2014). We found that there was a significant decrease in the expression of MLCK in IA by proteomic analysis. After interfering with MLCK RNA silencing, we found that SMC contractility decreased and apoptosis increased. In addition, SMA expression was downregulated. Therefore, we speculated that decreased MLCK could promote aneurysm development. Many other decreased proteins in IA and STA tissues, such as filamin-C, desmin, and aldehyde dehydrogenase, were found through proteomic analysis. We demonstrated that MLCK was less expressed in IASMCs and that other proteins were usually not expressed in SMCs. Therefore, we chose MLCK as a target in the current study.

There were also some limitations that need further improvement. IASMCs were isolated from IA tissues and cultured using SMCM. We isolated SMCs from the IA wall directly by cutting small pieces of IA tissues and only used cells within three passages to avoid the effects of *in vitro* culture conditions. The cells were derived with an apoptotic nature and could not be passed for several generations, thus limiting experiments *in vitro*. Whether the *mlck* gene plays a key role in IASMC dysfunction and whether *mlck* mutations exist still needs further exploration.

In summary, the present study provided novel evidence showing that the downregulation of MLCK in SMCs impacted the organization of mature and functional arteries. The lower expression of MLCK further led to increased apoptosis during the inflammatory response, which resulted in the loss of SMCs and contractile dysfunction. This study is the first to use primary IASMCs to detect SMC function. Our results provide further support that MLCK is involved in SMC contraction, proliferation and apoptosis. Our results also showed that the homeostasis of SMCs is crucial to the normal function of intracranial arteries.

## Author Contributions

YaS and PL designed and performed the experiments, analyzed the data, and drafted the manuscript and figures. G-YY and WZ conceived the project, designed the experiments, and edited the final manuscript. ZL and YuS participated in the study design. JH, SL, and YL contributed to specimen collection. ZZ and YW helped to design the experiments and interpreted the data.

## Conflict of Interest Statement

The authors declare that the research was conducted in the absence of any commercial or financial relationships that could be construed as a potential conflict of interest.

## Abbreviations

HBVSMC: human brain vascular smooth muscle cell
IA: intracranial aneurysm
IASMCs: intracranial aneurysm smooth muscle cells
MLCK: myosin light chain kinase
SMA: anti-SM-α actin
SMCs: smooth muscle cells
STA: superficial temporal artery
