# Supplementary material for: The Effect of Myosin Light Chain Kinase on the Occurrence and Development of Intracranial Aneurysm
Source: Front Cell Neurosci. 2018 Nov 13;12:416. doi: 10.3389/fncel.2018.00416 (PMC6282066; doi:10.3389/fncel.2018.00416)
Supplement: Supplementary file 1 [file Table_1.DOCX]

***Table 1 Characteristics of aneurysm in patients***

| **Sample no.** | **Sex** | **Age,y** | **Location of IA** | **Label-free** | **Western blot** | **immunostaining** |
| --- | --- | --- | --- | --- | --- | --- |
| 1 | M | 43 | MCA | + | - | - |
| 2 | M | 50 | ICA | + | - | - |
| 3 | F | 69 | MCA | + | - | - |
| 4 | F | 45 | ACA | + | - | - |
| 5 | F | 38 | PICA | + | - | - |
| 6 | F | 45 | MCA | - | - | + |
| 7 | F | 56 | MCA | - | - | + |
| 8 | F | 67 | PICA | - | + | - |
| 9 | F | 65 | MCA | - | + | - |
| 10 | F | 61 | MCA | - | + | - |

ACA, anterior carotid artery; ICA, internal carotid artery; MCA, middle cerebral artery; PICA, posterior inferior cerebellar artery; F, female; M, male. Label free proteomic, western blotting or immunostaining performed (+) or not performed (−).

***Characteristics of superficial temporal artery in patients***

| **Sample no.** | **Sex** | **Age,y** | **Label-free** | **Western blot** | **immunostaining** |
| --- | --- | --- | --- | --- | --- |
| 1 | F | 45 | + | - | - |
| 2 | M | 68 | + | - | - |
| 3 | F | 37 | + | - | - |
| 4 | F | 31 | + | - | - |
| 5 | M | 44 | + | - | - |
| 6 | M | 43 | - | - | + |
| 7 | F | 49 | - | - | + |
| 8 | M | 59 | - | + | - |
| 9 | F | 40 | - | + | - |
| 10 | F | 45 | - | + | - |

F, female; M, male. Label free proteomic, western blotting or immunostaining performed (+) or not performed (−).
